# Diagnostic Performance and Clinical Utility of the Uromonitor^®^ Molecular Urine Assay for Urothelial Carcinoma of the Bladder: A Systematic Review and Diagnostic Accuracy Meta-Analysis

**DOI:** 10.3390/diagnostics16020285

**Published:** 2026-01-16

**Authors:** Julio Ruben Rodas Garzaro, Anton Kravchuk, Maximilian Burger, Ingmar Wolff, Steffen Lebentrau, José Rubio-Briones, João Paulo Brás, Christian Gilfrich, Stephan Siepmann, Sascha Pahernik, Axel S. Merseburger, Axel Heidenreich, Matthias May

**Affiliations:** 1 Department of Urology, St. Elisabeth Hospital Straubing, Brothers of Mercy Hospital, 94315 Straubing, Germany; julio.rodas-garzaro@klinikum-straubing.de (J.R.R.G.); anton.kravchuk@klinikum-straubing.de (A.K.); christian.gilfrich@klinikum-straubing.de (C.G.); stephan.siepmann@klinikum-straubing.de (S.S.); 2 Department of Urology, Caritas St. Josef Medical Center, University of Regensburg, 93053 Regensburg, Germany; mburger@csj.de; 3 Department of Urology, University Medicine Greifswald, 17475 Greifswald, Germany; ingmar.wolff@med.uni-greifswald.de; 4 Department of Urology, Werner Forssmann Hospital, 16225 Eberswalde, Germany; steffen.lebentrau@klinikum-barnim.de; 5 Department of Urology, Hospital VITHAS 9 de Octubre, 46015 Valencia, Spain; jrubio@clinicadoctorrubio.es; 6 Instituto de Investigação e Inovação em Saúde (i3S), Universidade do Porto, 4200-135 Porto, Portugal; joao.bras@uromonitor.com; 7 Instituto de Patologia e Imunologia Molecular (Ipatimup), Universidade do Porto, 4200-135 Porto, Portugal; 8 Department of Urology, Nuremberg General Hospital, Paracelsus Medical University, 90419 Nuremberg, Germany; sascha.pahernik@klinikum-nuernberg.de; 9 Department of Urology, University Hospital Schleswig-Holstein, Campus Lübeck, 23538 Lübeck, Germany; axel.merseburger@uksh.de; 10 Department of Urology, Uro-Oncology, Robot-Assisted, and Specialized Urologic Surgery, University Hospital Cologne, 50937 Cologne, Germany; axel.heidenreich@uk-koeln.de

**Keywords:** urinary bladder neoplasms, transitional cell carcinoma, biomarkers, polymerase chain reaction, Uromonitor, urine cytology, sensitivity and specificity, predictive value of tests

## Abstract

**Background**: Urine cytology remains widely used for surveillance of non-muscle-invasive bladder cancer despite well-known limitations in sensitivity, especially for low-grade tumors. Uromonitor^®^, a molecular assay detecting TERT promoter, FGFR3, and KRAS mutations in voided urine, has emerged as a promising adjunct. To evaluate its suitability for routine use, a consolidated assessment of diagnostic performance and a direct comparison with urine cytology are needed. **Methods**: We conducted a prospectively registered systematic review (PROSPERO CRD420251173244), synthesizing all available studies that evaluated Uromonitor^®^ for the detection of urothelial carcinoma of the bladder (UCB). Methodological quality was assessed using the QUADAS-2 framework, and certainty of evidence was evaluated following GRADE for diagnostic tests. Sensitivity was prespecified as the primary endpoint. Comparative datasets were identified, and random-effects meta-analyses were performed for sensitivity, specificity, accuracy, and predictive values (PVs). **Results**: Across eight cohorts evaluating Uromonitor^®^, 832 of 3196 patients (26.0%) had histologically confirmed UCB. Aggregated sensitivity was 0.55 (95% CI 0.52–0.58). Specificity was 0.95 (0.94–0.96). Accuracy was 0.85 (0.83–0.86). PPV was 0.79 (0.76–0.82), and NPV was 0.86 (0.84–0.87). Across seven paired datasets, urine cytology demonstrated a sensitivity of 0.42, a specificity of 0.91, an accuracy of 0.78, a PPV of 0.64, and an NPV of 0.81. Pooled odds ratio for sensitivity was 3.16 (0.73–13.76), while diagnostic accuracy yielded 1.71 (1.01–2.90). Differences in specificity and NPV were not statistically significant, whereas the PPV favored Uromonitor^®^, reaching statistical significance in pooled analyses. **Conclusions**: Uromonitor^®^ demonstrates higher sensitivity and improved accuracy compared with urine cytology, although current performance remains insufficient for stand-alone surveillance. The sensitivity estimate showed very low certainty due to pronounced heterogeneity, underscoring the need for careful interpretation. With advancing DNA recovery methods, incorporation of droplet digital PCR, and rigorous evaluations in prospective multicenter studies, Uromonitor^®^ may become an integral element of risk-adapted follow-up strategies.

## 1. Introduction

Non-muscle-invasive bladder cancer (NMIBC) is one of the most common malignancies of the urinary tract and accounts for nearly 70% of new diagnoses [1]. Although most tumors are detected at an early stage, NMIBC tends to recur and carries a sustained risk of progression, creating a substantial and often long-term burden for patients and health care systems [1,2]. International guidelines therefore mandate intensive cystoscopic surveillance throughout follow-ups, a strategy that is oncologically sound but invasive, uncomfortable, and increasingly costly [1,2,3]. The need for reliable non-invasive biomarkers that could safely reduce the frequency of these procedures has consequently become more pressing [1,2,3,4,5,6,7].

Urine cytology remains the most widely used adjunct in routine surveillance [1,2,3]. Its specificity is excellent, but its sensitivity for low-grade disease is limited, and its performance is affected by specimen adequacy, interobserver variability, and institutional expertise [1,2,3,4,5,6,7]. These constraints have encouraged the development of molecular assays that detect tumor-specific genomic alterations in exfoliated urothelial cells, including RNA-based and DNA-based platforms. Among DNA based approaches, assays that target hotspot mutations in the TERT promoter, FGFR3 and, more recently, KRAS have gained particular attention. These alterations represent biologically meaningful drivers of urothelial carcinogenesis and can be detected even when cytological atypia is subtle or absent [8].

Uromonitor^®^ is a quantitative real-time polymerase chain reaction assay designed to identify these mutations in voided urine. The first generation of the assay (Version 1) interrogates TERT and FGFR3, whereas the current generation (Version 2) incorporates KRAS to broaden genomic coverage and potentially improve diagnostic sensitivity. Early analytical studies demonstrated that these aberrations persist in urinary tumor DNA and can be detected at very low allelic fractions with high technical fidelity [9,10]. Subsequent clinical evaluations showed greater sensitivity than urine cytology, particularly for low-grade recurrences, while maintaining high specificity [11,12,13]. However, the evidence base remains heterogeneous with respect to assay generation, study design, recurrence definitions, reference standards, and patient characteristics, which limits the ability to derive comparative accuracy estimates with sufficient methodological rigor [14,15,16].

Because timely detection of recurrence is central to preserving favorable oncologic outcomes and procedural burden continues to rise, a contemporary reassessment of Uromonitor^®^ is warranted. Our previous systematic review included 1190 test evaluations from four studies, only two of which permitted direct comparison with urine cytology and therefore supported quantitative synthesis [8]. Several additional investigations, including real world multicenter datasets, have since been published.

The objective of this systematic review and meta-analysis was to integrate all current evidence across assay generations, with sensitivity prespecified as the primary clinical endpoint. Secondary objectives included pooled analyses of specificity, diagnostic odds ratios, predictive values, and likelihood ratios, together with direct comparisons of Uromonitor^®^ and urine cytology using identical patient level datasets. This consolidated synthesis provides an updated and clinically relevant evidence base to inform future surveillance strategies for patients with NMIBC.

## 2. Methods

### 2.1. Study Design and Protocol Registration

We conducted a systematic review and diagnostic test accuracy meta-analysis to evaluate the performance of the Uromonitor^®^ assay compared with urine cytology for detecting urothelial carcinoma of the bladder (UCB). The review adhered to the Preferred Reporting Items for Systematic Reviews and Meta-Analyses of Diagnostic Test Accuracy Studies (PRISMA-DTA) and the Cochrane Handbook for Diagnostic Test Accuracy Reviews and methodological guidance [17,18,19]. The protocol was prospectively registered in PROSPERO (CRD420251173244) and followed the predefined objectives, eligibility criteria, and analytic framework.

The primary outcome was sensitivity, defined as the proportion of histologically confirmed UCB correctly identified by Uromonitor^®^ or urine cytology. Sensitivity was selected a priori because minimizing missed recurrences represents the central clinical imperative in NMIBC. Secondary outcomes included specificity, positive predictive value (PPV), negative predictive value (NPV), and overall diagnostic accuracy. Diagnostic accuracy was evaluated separately for Uromonitor^®^ Version 1 and Version 2. Comparative meta-analyses were restricted to studies that assessed both Uromonitor^®^ and urine cytology within the same patient cohorts.

### 2.2. Search Strategy and Study Selection

A comprehensive search of MEDLINE (via PubMed), Embase, Web of Science, Scopus, and the Cochrane Library was conducted from database inception through November 2025. The search strategy combined controlled vocabulary and free-text terms capturing the index test, its molecular targets and the clinical context of urothelial carcinoma of the bladder. No language, geographical, or temporal restrictions were applied. The full electronic search strategy used in MEDLINE was: (“Uromonitor” OR “Uromonitor test” OR “TERT promoter mutation” OR “FGFR3 mutation” OR “KRAS mutation”) AND (“urine” OR “urinary”) AND (“bladder cancer” OR “urothelial carcinoma”) AND (“diagnostic accuracy” OR “sensitivity” OR “specificity” OR “cytology”).

This strategy was adapted as appropriate for Embase, Web of Science, Scopus, and the Cochrane Library. After removal of duplicates, two investigators (J.R.R.G. and A.K.) independently screened titles and abstracts, each blinded to the other’s assessments. Full texts of potentially eligible studies were retrieved and evaluated against the predefined criteria in the PROSPERO protocol. Studies were eligible if they

1. Evaluated Uromonitor^®^ Version 1 or Version 2;
2. Reported sufficient data to construct two-by-two contingency tables for at least one diagnostic endpoint;
3. Used histopathology as the reference standard;
4. Enrolled adults with suspected or confirmed urothelial carcinoma of the bladder.

Any disagreements were resolved by consensus adjudication with the senior investigator (M.M.).

### 2.3. Data Extraction and Index/Comparator Test Definitions

A standardized extraction template was piloted before full implementation. Two reviewers (J.R.R.G. and A.K.) independently extracted data on study design, clinical setting, assay version, mutation targets, cytology thresholds, reference standards, recurrence definitions, specimen handling, and all diagnostic outcomes. True positive (TP), false positive (FP), true negative (TN), and false negative (FN) values were collected exactly as reported.

If contingency data were incomplete, corresponding authors were contacted for clarification. The CUETO group provided additional cytology data that allowed inclusion of their study in the comparative synthesis [14]. Conflicts in extraction were resolved by consensus with oversight from the senior investigator (M.M.).

Urine cytology results were dichotomized according to the Paris System, with categories negative for high-grade malignancy classified as test negative and categories suspicious or positive for high-grade disease classified as test positive [1,2,3]. Uromonitor^®^ was considered positive when at least one of the target mutations (TERT promoter, FGFR3, or KRAS) was detected [8]. These definitions were applied consistently across all included studies to ensure methodological comparability.

### 2.4. Risk of Bias and Applicability Assessment

Study-level risk of bias and concerns regarding applicability were assessed using the QUADAS-2 tool [20]. Both reviewers (J.R.R.G. and A.K.) independently evaluated the four domains of patient selection, index test, reference standard, and flow and timing. Each domain was graded as low, high, or unclear risk. Applicability concerns were assessed in a corresponding manner with respect to the target population, the conduct and interpretation of the index test, and the appropriateness of the reference standard. Blinding of index and reference test interpretation was evaluated as part of the QUADAS-2 assessment and extracted whenever explicitly reported in the primary studies.

Any discrepancies between both reviewers were discussed and resolved by the senior investigator (M.M.), who provided the final consensus interpretation. Certainty of evidence was assessed using the GRADE framework for diagnostic test accuracy [21]. Judgments regarding risk of bias, inconsistency, indirectness, imprecision, and publication bias contributed to the certainty rating for each endpoint.

### 2.5. Data Preparation and Statistical Analysis

Two-by-two contingency tables were constructed for each diagnostic endpoint using standard definitions. Sensitivity, specificity, positive predictive value (PPV), negative predictive value (NPV), and overall diagnostic accuracy were calculated together with exact binomial 95% confidence intervals (CI). As prespecified, all available Uromonitor^®^ data were pooled for the primary analyses to maximize statistical power across generations. To ensure methodological consistency with the current assay configuration, sensitivity analyses were performed restricting pooling to Uromonitor^®^ Version 2 (combining TERT, FGFR3, and KRAS mutation detection). Forest plots were constructed for all diagnostic metrics.

Receiver-operating-characteristic (ROC) curves were generated by relating sensitivity to 1 minus specificity using binary test outcomes as predictors and histologically confirmed UCB as the reference standard. Corresponding area-under-the curve (AUC) values quantify the overall diagnostic information content of each test, with *p*-values reflecting their statistical separation from chance performance (defined as an AUC of 0.5). Because all included studies reported strictly binary outcomes for both index and comparator tests, HSROC modeling was not applied, as it requires variability in diagnostic thresholds across studies.

Comparative pooling was limited to studies that reported paired results for both Uromonitor^®^ and urine cytology. Odds ratios (OR) were selected a priori as the preferred effect measure, since they are independent of disease prevalence, allow symmetric modeling across tests, and represent the recommended metric in comparative diagnostic accuracy research. An OR greater than 1 indicates superior performance of Uromonitor^®^ over urine cytology for the respective endpoint. Random-effects models were fitted using restricted maximum likelihood estimation, as variations across clinical settings were anticipated. Standard errors and confidence intervals were stabilized using the Knapp–Hartung adjustment. For interpretability, pooled log-OR values were exponentiated and reported as OR.

Between-study heterogeneity was evaluated using Cochran’s Q, τ^2^, and the I^2^ statistic. Interpretation focused on I^2^ because it conveys the proportion of observed variability attributable to genuine differences rather than sampling error. Predefined qualitative thresholds were applied, with values below 25% interpreted as low inconsistency, 25–50% as moderate, and greater than 50% as substantial heterogeneity.

Potential publication bias was assessed using the two-sided Harbord regression asymmetry test, which provides a refined alternative to the Egger test for odds ratio outcomes. A statistically significant *p*-value suggests small-study effects or possible publication bias, whereas a non-significant result strengthens the confidence in absence of such distortions.

All analyses were conducted using SPSS Statistics Version 31.0 (IBM Corp., Armonk, NY, USA), with all tests interpreted as two-sided whenever applicable, and a type I error threshold of *p* ≤ 0.05 was considered indicative of statistical significance.

## 3. Results

### 3.1. Study Selection and Study Characteristics

**Figure 1:**
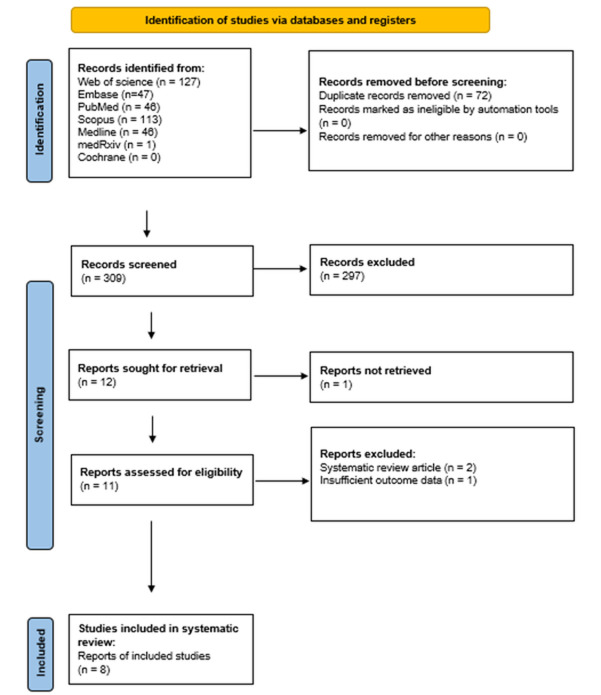
PRISMA flow diagram of study selection. Flow diagram outlining identification, screening, eligibility assessment and inclusion of studies according to PRISMA guidelines.

The systematic search identified eight diagnostic-accuracy cohorts evaluating the Uromonitor^®^ assay for detection of UCB in patients undergoing evaluation for hematuria or surveillance for NMIBC. Seven of the eight cohorts reported paired results for Uromonitor^®^ and urine cytology [9,10,11,13,14,15,16], whereas one cohort contributed Uromonitor^®^ data only [12]. The complete study selection process is depicted in Figure 1, and study characteristics are summarized in Table 1. One study applied the Uromonitor^®^ assay as Version-1 (only TERT + FGFR3 mutation-analytic) and subsequently implemented Version-2 in a subgroup of patients [9].

Across all eight datasets, 3196 patients underwent Uromonitor^®^ testing, with cohort sizes ranging from 97 to 1145 (Table 1). Histologically confirmed UCB was present in 832 patients, corresponding to an overall prevalence of 26.0%. The combined population encompassed both diagnostic and surveillance settings and included low-grade and high-grade disease as well as non-muscle- and muscle-invasive tumor stages. Detailed eligibility criteria, reference standards, and full contingency data for both Uromonitor^®^ and cytology are presented in Table 1.

Risk of bias was moderate overall. Using the QUADAS 2 assessment, three studies had low risk of bias across all domains, whereas four exhibited unclear or high-risk in-patient selection or in flow and timing. Interpretation of the index test was generally blinded to histopathology, though blinding to the comparator test was not consistently documented. Applicability concerns reflected heterogeneity in clinical pathways and pretest probabilities (Table 2).

### 3.2. Aggregate Diagnostic Performance of Uromonitor^®^ and Urine Cytology

Across all eight Uromonitor^®^ cohorts, the pooled sensitivity for detecting histologically confirmed UCB was 0.55 (95% CI: 0.52 to 0.58), with a specificity of 0.95 (0.94 to 0.96), a PPV of 0.79 (0.76 to 0.82), an NPV of 0.86 (0.84 to 0.87), and an overall accuracy of 0.85 (0.83 to 0.86) (Table 3). In absolute terms, Uromonitor^®^ yielded 459 true positives, 2242 true negatives, 122 false positives, and 373 false negatives across 3196 tests (Table 1). Supplementary Figure S1 illustrates the potential net clinical benefit, projected per 1000 Uromonitor^®^ tests, in terms of avoided missed recurrences and unnecessary cystoscopic evaluations.

Among the seven cohorts with head-to-head comparisons, comprising 2562 patients with available urine cytology results, 685 of whom had confirmed disease (26.7%), urine cytology demonstrated a sensitivity of 0.42, a specificity of 0.91, a PPV of 0.64, an NPV of 0.81, and an overall accuracy of 0.78 (Table 3). These values correspond to 291 true positives, 1714 true negatives, 163 false positives, and 394 false negatives.

Taken together, Uromonitor^®^ increased absolute sensitivity by 12.7% points compared with cytology and demonstrated higher overall accuracy, an improvement of 6.3% points, and a markedly higher PPV, an improvement of 14.9% points. Specificity and NPV remained broadly comparable, with differences of 3.5% and 4.4% points, respectively. These aggregated findings define the clinical context for the subsequent meta-analytic comparisons.

The AUC values of the Uromonitor^®^ assay across eight studies and of urine cytology across seven studies were 0.750 and 0.669, respectively, with *p*-values < 0.001 for both comparisons against chance performance (Table 3).

Supplementary Table S1 reports the aggregated sensitivities of the Uromonitor^®^ assay and urine cytology from the eight studies that provided stage-stratified results for NMIBC and MIBC, grade-stratified results for LG and HG tumors, and separate data for primary and recurrent disease.

### 3.3. Meta-Analytic Comparison of Uromonitor^®^ and Urine Cytology

#### 3.3.1. Primary Outcome: Sensitivity

**Figure 2:**
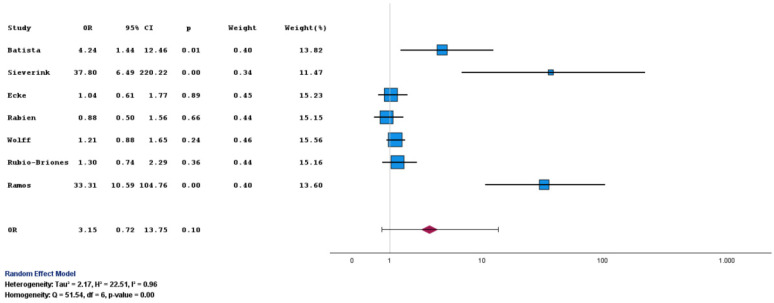
Forest plot of sensitivity for Uromonitor^®^ versus urine cytology. Forest plot comparing sensitivity estimates for Uromonitor^®^ and urine cytology across the included studies [9,10,11,13,14,15,16].

Sensitivity was the prespecified primary endpoint. Using all original datasets, including the earliest cohort that employed Version 1 of the assay [9], Uromonitor^®^ showed higher, although not statistically significant, odds of detecting UCB compared with cytology, with a pooled OR of 3.15 (95% CI: 0.72 to 13.75; *p* = 0.10) (Table 3 and Figure 2).

##### Sensitivity Analyses: Harmonized Version 2 Assay

After recalculating the earliest cohort with the harmonized Version 2 assay used in later studies, the pooled OR for sensitivity was 4.26 (95% CI: 0.75 to 24.29; *p* = 0.09) (Table 3 and Supplementary Figure S2). Despite a modest change in precision, the point estimate remained essentially unchanged.

#### 3.3.2. Secondary Outcomes: Specificity, PPV, NPV, and Accuracy

**Figure 3:**
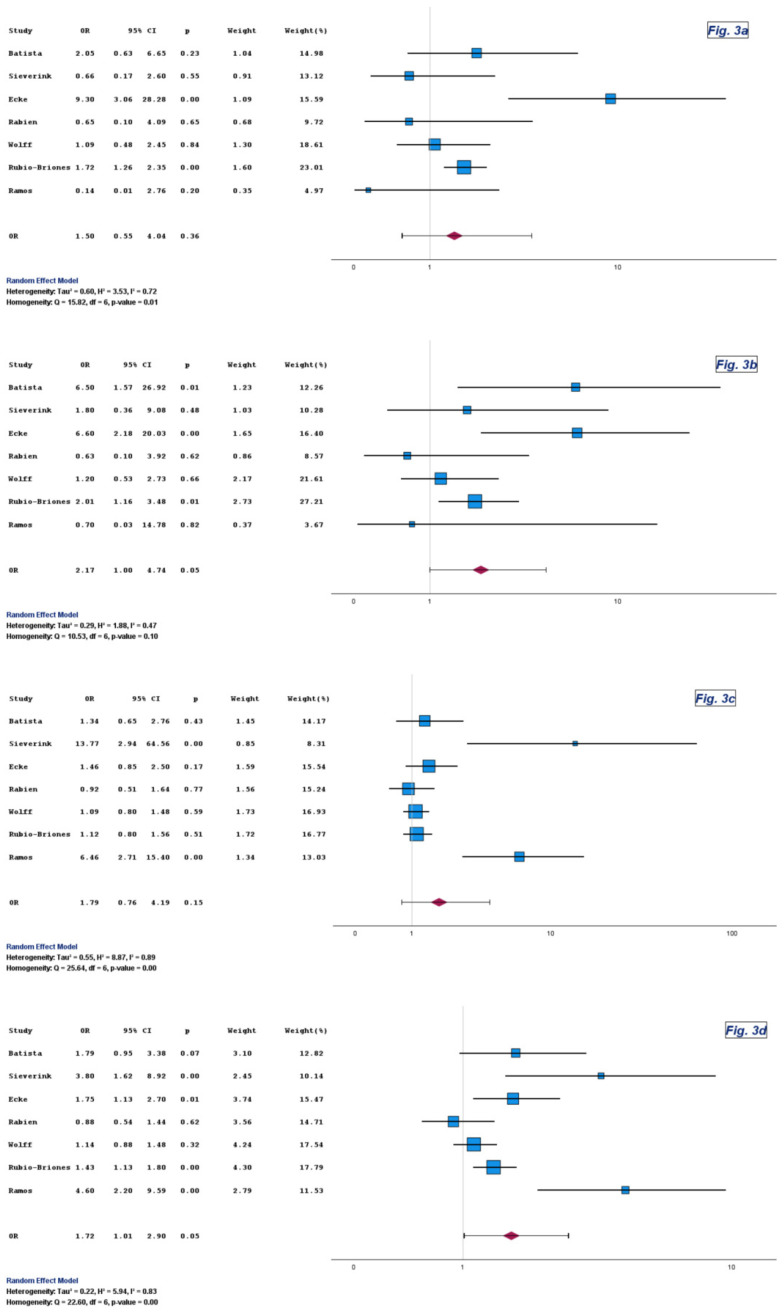
Forest plots of specificity (**a**), PPV (**b**), NPV (**c**), and overall diagnostic accuracy (**d**) for Uromonitor^®^ versus urine cytology. Forest plots comparing specificity, positive predictive value, negative predictive value, and overall diagnostic accuracy for Uromonitor^®^ and urine cytology across the included studies [9,10,11,13,14,15,16].

Specificity did not differ materially between Uromonitor^®^ and urine cytology. Pooled ORs were close to unity with wide CIs in both Version 1 and Version 2 analyses (Table 3, Figure 3 and Figure S3).

The pooled estimates for PPV reached statistical significance in favor of Uromonitor^®^, with ORs of 2.17 and 2.04 and corresponding *p*-values of 0.05 and 0.04, respectively (Table 3, Figure 3 and Figure S3).

The pooled ORs for NPV also favored Uromonitor^®^ in both Version 1 and Version 2, although without statistical significance, with ORs of 1.79 and 2.11 and corresponding *p*-values of 0.15 and 0.10, respectively (Table 3, Figure 3 and Figure S3).

Uromonitor^®^ consistently outperformed urine cytology with respect to overall diagnostic accuracy. The corresponding pooled ORs were 1.72 (95% CI: 1.01 to 2.90; *p* = 0.05) and 1.77 (95% CI: 1.03 to 3.07; *p* = 0.04) (Table 3, Figure 3 and Figure S3). Although CIs occasionally approached unity, the direction and magnitude of effect were stable across assay generations.

### 3.4. Certainty of Evidence

Using the GRADE approach, the certainty of evidence for the primary endpoint, sensitivity, was judged to be very low. This rating reflects serious concerns regarding risk of bias, serious imprecision due to wide confidence intervals around the pooled odds ratio, and evidence of publication bias, whereas inconsistency and indirectness were not considered serious (Supplementary Table S2). The meta-analysis for sensitivity showed substantial between-study heterogeneity, with I^2^ values of 96% and 97% in the overall and Version 2 only analyses, respectively, accompanied by wide confidence intervals that further reduced confidence in the effect estimate.

In contrast, the certainty of evidence for specificity, PPV, and overall diagnostic accuracy was rated moderate. For these outcomes, risk of bias, inconsistency, and indirectness were not deemed serious, and the certainty was downgraded only for imprecision, which was classified as borderline for PPV and accuracy. For NPV, imprecision was judged serious, resulting in a low certainty rating despite otherwise acceptable methodological quality.

Supplementary Table S3 summarizes the results of the Harbord regression asymmetry test used to assess potential publication bias. Statistically significant asymmetry was observed for sensitivity in both the overall pooled analysis and the Version 2 only analysis (*p* = 0.028 and *p* = 0.012, respectively), consistent with a possible small study effect for this endpoint. In contrast, all remaining diagnostic metrics, including specificity, PPV, NPV, and accuracy across both analytic settings, showed no statistically significant asymmetry (all *p* values greater than 0.05), which argues against relevant publication bias for these parameters.

## 4. Discussion

This systematic review and diagnostic accuracy meta-analysis, comprising eight cohorts and 3196 assessments including 832 histologically confirmed cases of urothelial carcinoma of the bladder (UCB), demonstrates that Uromonitor^®^ provides a consistent and biologically plausible improvement in tumor detection compared with urine cytology. Across heterogeneous real-world diagnostic pathways, the assay achieved higher overall diagnostic accuracy and substantially stronger positive predictive values. The absolute gain of 13% points in sensitivity, resulting in a pooled sensitivity of 55% and a negative predictive value (NPV) of 86%, represents a clinically relevant increment. At the same time, an NPV of 86% indicates that approximately one in seven patients with UCB would receive a false-negative result, underscoring that the assay cannot be used as a stand-alone surveillance tool in non-muscle-invasive bladder cancer (NMIBC). The stability of diagnostic performance across assay generations, despite pronounced heterogeneity suggests that the observed effect reflects a genuine genomic signal rather than statistical fluctuation. Collectively, these findings support Uromonitor^®^ as an adjunctive marker capable of strengthening established surveillance strategies, while highlighting the need for harmonized implementation protocols and rigorously designed prospective validation before broader clinical adoption.

The molecular rationale underlying these findings is coherent. Uromonitor^®^ targets recurrent mutations in the TERT promoter, FGFR3, and KRAS, which represent early, clonally stable, and biologically relevant driver alterations in NMIBC [22,23]. Their persistence facilitates molecular detection even when cellular yield is low or morphological atypia remains below cytological thresholds [24]. By contrast, urine cytology relies on subjective interpretation and exhibits limited sensitivity for low-grade recurrences, which account for the majority of false-negative results [25]. These mechanistic considerations align closely with the consistently superior diagnostic performance observed in the present synthesis.

From a clinical perspective, two implications emerge. In the diagnostic work-up of hematuria, mutation-based assays may assist in prioritizing patients for cystoscopy and cross-sectional imaging, particularly in settings with constrained procedural capacity. In NMIBC surveillance, the combined increase in sensitivity and the directionally improved NPV indicate that recurrence can be excluded with greater confidence in patients with repeatedly negative molecular findings. In carefully selected low-risk individuals, this may support cautious extension of cystoscopic intervals within a shared decision-making framework, provided that no concurrent clinical risk factors are present. Importantly, negative molecular results cannot replace cystoscopy and should not be interpreted as definitive evidence of disease absence. Conversely, persistent mutation positivity in the absence of visible tumor warrants intensified diagnostic evaluation, including enhanced cystoscopy or multimodal imaging, given the biological plausibility of molecularly detectable recurrence preceding macroscopic disease. These observations support integration of molecular diagnostics into risk-adapted surveillance models without challenging the central oncologic role of cystoscopy for inspection, resection, and histopathological confirmation [1,2,3,24,25,26,27,28,29].

To place these findings into a broader diagnostic context, contemporary urine-based bladder diagnostic tests can be conceptually categorized into protein-based assays (including Nuclear Matrix Protein 22^®^, Bladder Tumor Antigen^®^, ADXBladder^®^, and cytokeratin-based tests), cell-based approaches (such as UroVysion^®^ and CellDetect^®^), RNA-based assays (including Cxbladder Monitor^®^, Xpert BC^®^, and microRNA panels), and DNA-based tests, which encompass methylation-based platforms and mutation-focused assays such as Uromonitor^®^ [1,2,3]. Current European Association of Urology guidelines for NMIBC surveillance emphasize urine cytology and summarize the evidence for molecular multiplex urine marker tests, while not recommending their routine use [1]. In alignment with this framework, we provide an overview of reported diagnostic performance metrics for molecular multiplex urine marker assays used in surveillance, as summarized in Supplementary Table S4. This table is structured analogously to the corresponding guideline summary and highlights substantial methodological heterogeneity, differences in patient selection, and the absence of direct head-to-head comparisons, which preclude definitive comparative conclusions [1].

The discrepancy between the present findings and our earlier meta-analysis warrants careful consideration. The prior synthesis, which included four studies and 1190 Uromonitor^®^ evaluations, reported a pooled sensitivity of 80.2%, an NPV of 96.6%, and an overall diagnostic accuracy of 94.5% [8]. In the current analysis, sensitivity declined by approximately 25% points, and both NPV and accuracy were reduced by about 10% points. This divergence likely reflects the higher disease prevalence in the present dataset (26.0% versus 14.9%) and the inclusion of two large multicenter real-world cohorts with comparatively lower diagnostic performance [13,14]. Exclusion of these datasets would yield estimates closer to those reported previously. In the German multicenter study by Wolff et al., all genomic analyses were performed centrally at the original Portuguese reference laboratory [13], whereas in the Spanish CUETO study by Rubio-Briones et al., only the initial evaluation phase was centralized and subsequent analyses were conducted locally [14]. Although the impact of pre-analytical and logistical variation cannot be quantified precisely, the complexity of specimen collection, processing, and transport in multicenter real-world environments represents a plausible contributor to heterogeneity. Differences in transport time, cooling conditions, and urine filtration may further influence mutation yield. Compared with our 2023 review [8], the present analysis incorporates a substantially larger and more contemporary evidence base generated under routine surveillance conditions, thereby providing a more clinically representative appraisal of assay performance. While pooled sensitivity was rated as very low certainty using GRADE, this assessment primarily reflects methodological heterogeneity driven by pragmatic implementation rather than an intrinsic biological limitation of the assay.

Interpretation of these findings should also consider the broader landscape of urine-based diagnostics summarized in contemporary guidelines. Although multiplex urine markers are not recommended for routine NMIBC surveillance, the EAU acknowledges strong diagnostic performance for established platforms such as Xpert BC^®^ Monitor and Bladder EpiCheck™ [1]. A recent systematic review including six studies and 1588 urinary assessments reported a sensitivity of 81% and an NPV of 94% for Bladder EpiCheck™ [28]. Such benchmarks define the operational threshold toward which mutation-based assays like Uromonitor^®^ must advance before routine clinical implementation can be considered. While methylation- and transcript-based assays interrogate broader epigenetic or transcriptional landscapes, mutation-based tests offer distinct biological advantages, including the early occurrence and clonal stability of TERT promoter alterations, which support detectability even in samples with limited cellularity. These complementary features suggest that further refinement of mutation-focused assays remains warranted.

Several limitations merit consideration. The number of eligible diagnostic cohorts was limited, restricting precision in subgroup analyses and widening confidence intervals for secondary endpoints. Between-study heterogeneity was substantial, with I^2^ values approaching 96% for sensitivity and 97% in analyses restricted to version 2 datasets. This likely reflects genuine variability in diagnostic context, recurrence definitions, and pre-analytical workflows rather than random error. Sensitivity demonstrated statistically significant asymmetry in the Harbord regression for both pooled and version 2 datasets, consistent with a small-study effect. No evidence of publication bias was detected for specificity, positive predictive value, NPV, or overall diagnostic accuracy. Analyses were based on aggregated rather than individual patient data, limiting exploration of heterogeneity. In addition, a formal cost-effectiveness analysis was beyond the scope of this review, as test-related costs and reimbursement structures for molecular urine assays and urine cytology vary substantially across healthcare systems, thereby limiting the generalizability of economic comparisons. Blinding of laboratory assessment was not consistently reported, introducing potential interpretative bias. All included studies were conducted in Europe, underscoring the need for validation in other geographical regions. Importantly, this review was prospectively registered and adhered fully to PRISMA and PRISMA-DTA recommendations, with compliance documented in Supplementary Tables S5 and S6.

Future research should prioritize multicenter evaluations embedded within standardized surveillance frameworks and predefined diagnostic pathways. Such studies should quantify the impact of molecular testing on clinical decision-making, surveillance intensity, and cost-effectiveness. Integration of patient-reported outcomes will be essential to assess acceptability and anxiety associated with uncertain molecular findings. Decision curve analysis may aid determination of threshold probabilities at which Uromonitor^®^ confers net clinical benefit. Optimization of pre-analytical workflows, including urine filtration, nucleic acid recovery, and adoption of droplet digital PCR, may further enhance sensitivity [16,30]. Combined biomarker strategies integrating mutation, methylation, and transcriptomic profiling may extend diagnostic reach, particularly for carcinoma in situ and upper tract involvement.

## 5. Conclusions

Uromonitor^®^ improves detection of urothelial carcinoma of the bladder compared with urine cytology by increasing sensitivity while maintaining high specificity and providing a modest but clinically meaningful gain in negative predictive value. These findings support its role as an adjunctive tool that can refine surveillance strategies in patients with non-muscle-invasive disease. Cystoscopy remains indispensable for visual assessments, tissue acquisition, and histopathological confirmation; however, Uromonitor^®^ offers a molecular complement that may inform risk-adapted follow-ups. Further optimization of tumor DNA recovery, integration of multimodal biomarker approaches, and validation in prospective multicenter studies are required to advance toward more accurate and potentially less invasive surveillance pathways.

## Figures and Tables

**Table 1:** Characteristics of the included diagnostic accuracy cohorts. Summary of eight diagnostic accuracy studies evaluating the Uromonitor^®^ assay for detecting urothelial carcinoma of the bladder. Reported variables include study period, design, assay generation, sample size, number of histologically confirmed cases, and the distribution of primary and recurrent tumors. Key clinicopathological features, including rates of high-grade and muscle-invasive disease, are presented together with diagnostic contingency data for Uromonitor^®^ and urine cytology (true positives, true negatives, false positives, and false negatives).

Study	Study Period	Study Design	UM Version	N with UCB/N (%)	Prim./Rec. UCB (%)	HG UCB (%)	T2 UCB (%)	TP UM/UC	TN UM/UC	FP UM/UC	FN UM/UC
Batista [9]	2014–2017	Prospective, multicenter, observational diagnostic validation study	v1 + v2	62/185 (33.5%)	28 (45.1%)/ 34 (54.8%)	24 (38.7%)	2 (3.2%)	39/6	117/57	6/6	23/15
Sieverink [10]	2016–2018	Prospective case–control single-center	v2	29/97 (29.9%)	0/ 29 (100%)	12 (41.4%)	0	27/5	59/30	9/3	2/14
Ecke [15]	2021–2023	Prospective multicenter diagnostic accuracy study	v2	110/187 (58.8%)	0/ 110 (100%)	36 (32.7%)	14 (12.7%)	63/62	73/51	4/26	47/48
Rabien [16]	2021–2023	Prospective case–control single-center	v2	94/142 (66.2%)	74 (78.7%)/ 20 (21.3%)	29 (30.8%)	21 (22.3%)	47/50	45/46	3/2	47/44
Wolff [13]	2019–2024	Prospective, multicenter, double-blind real-world diagnostic accuracy study	v2	339/532 (63.7%)	248 (46.6%)/ 91 (17.1%)	186 (54.9%)	60 (11.3%)	167/128	180/153	13/12	172/159
Rubio-Briones [14]	2024	Prospective multicenter real-world observational validation study	v2	112/1145 (9.8%)	0/ 112 (100%)	542/1178 (46.0%)	0	40/32	962/896	71/114	72/75
Ramos [11]	2020–2022	Prospective single-center observational diagnostic validation study	v2	47/528 (8.9%)	0/ 47 (100%)	41 (87.2%)	2 (4.2%)	41/8	478/481	3/0	6/39
Azawi * [12]	2019–2021	Prospective, multicenter, observational surveillance study	v2	39/380 (10.3%)	0/ 39 (100%)	n.a.	n.a.	35/n.a.	328/n.a.	13/n.a.	4/n.a.

**Legend:** FN UM/UC: false negatives based on test results of Uromonitor^®^ and urine cytology; FP UM/UC: false positives based on test results of Uromonitor^®^ and urine cytology; HG UCB (%): proportion of high-grade urothelial carcinoma of the bladder; N with UCB/N (%): number of patients with histologically confirmed urothelial carcinoma of the bladder out of the total cohort, expressed as absolute count and percentage; n.a.: not available; Prim./Rec. UCB (%): proportion of primary versus recurrent urothelial carcinoma of the bladder; Study design: design of the respective diagnostic study; Study period: timeframe during which the study was conducted; T2 UCB (%): proportion of stage T2 urothelial carcinoma of the bladder; TN UM/UC: true negatives based on test results of Uromonitor^®^ and urine cytology; TP UM/UC: true positives based on test results of Uromonitor^®^ and urine cytology; UC: urine cytology; UM: Uromonitor^®^ molecular urine assay; UM version: assay generation of Uromonitor^®^ used in the respective study (Version 1 or Version 2). * Azawi et al. [12] did not report urine cytology and therefore contributed only to single-arm aggregated analyses.

**Table 2:** Risk of bias and applicability concerns (QUADAS 2). Methodological assessment of the included studies using the QUADAS 2 framework, with judgments for patient selection, index test, reference standard, and flow and timing, together with evaluations of applicability to the review question.

Study	Patient Selection (Risk)	Index Test (Risk)	Reference Standard (Risk)	Flow and Timing (Risk)	Patient Selection (Applicability)	Index Test (Applicability)	Reference Standard (Applicability)
Batista [9]	Low risk	Low risk	Low risk	Low risk	Low concern	Low concern	Low concern
Sieverink [10]	Low risk	Low risk	Unclear	Unclear	Low concern	Low concern	Low concern
Ecke [15]	Low risk	Low risk	Unclear	Unclear	Low concern	Low concern	Low concern
Rabien [16]	Low risk	Low risk	Low risk	Unclear	Low concern	Low concern	Low concern
Wolff [13]	Low risk	Low risk	Unclear	Low risk	Low concern	Low concern	Low concern
Rubio-Briones [14]	Low risk	Low risk	High risk	Unclear	Low concern	Low concern	Low concern
Ramos [11]	Low risk	Low risk	High risk	Low risk	Low concern	Low concern	Low concern
Azawi [12]	High risk	Low risk	High risk	Unclear	Low concern	Low concern	Low concern
Summary plot							
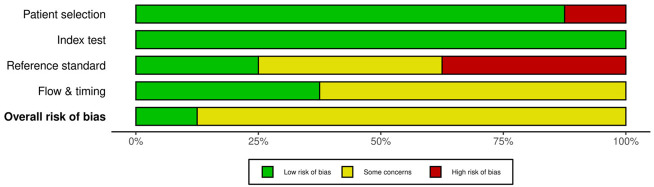							

**Table 3:** Summary of diagnostic accuracy outcomes for Uromonitor^®^ and urine cytology. Study level and pooled diagnostic indices for Uromonitor^®^ and urine cytology, including sensitivity, specificity, positive predictive value, negative predictive value, accuracy, and diagnostic odds ratios. Aggregated proportions based on raw counts are shown for Uromonitor^®^ (eight studies) and urine cytology (seven studies).

Diagnostic Parameter	Uromonitor^®^ (8 Studies)	Urine Cytology (7 Studies)	Difference	Comparative OR (95% CI), *p* UM (Version 1 + 2) vs. UC	Comparative OR (95% CI), *p* UM (Version 2) vs. UC
**Sensitivity** (95% CI)	0.552 (0.518–0.585)	0.425 (0.388–0.462)	Δ = 0.127 (0.077–0.177)	3.15 (0.72–13.75), *p* = 0.10	4.26 (0.75–24.29), *p* = 0.09
**Specificity** (95% CI)	0.948 (0.939–0.957)	0.913 (0.900–0.925)	Δ = 0.035 (0.020–0.051)	1.50 (0.55–4.04), *p* = 0.36	1.17 (0.40–3.45), *p* = 0.73
**PPV** (95% CI)	0.790 (0.755–0.821)	0.641 (0.596–0.684)	Δ = 0.149 (0.094–0.204)	2.17 (1.00–4.74), *p* = 0.05	2.04 (1.03–4.06), *p* = 0.04
**NPV** (95% CI)	0.857 (0.843–0.870)	0.813 (0.796–0.829)	Δ = 0.044 (0.023–0.066)	1.79 (0.76–4.19), *p* = 0.15	2.11 (0.82–5.43), *p* = 0.10
**Accuracy** (95% CI)	0.845 (0.833–0.856)	0.783 (0.766–0.798)	Δ = 0.063 (0.042–0.083)	1.72 (1.01–2.90), *p* = 0.05	1.77 (1.03–3.07), *p* = 0.04
**AUC** (95% CI), *p*	0.750 (0.729–0.771), *p* < 0.001	0.669 (0.644–0.694), *p* < 0.001	Δ = 0.081 (0.049–0.113)	-	-

**Legend:** AUC: area under the curve; CI: confidence interval; Δ: absolute difference; NPV: negative predictive value; OR: odds ratio; PPV: positive predictive value; UC: urine cytology; UM: Uromonitor^®^.

## Data Availability

The datasets generated and analyzed during the present study are not publicly available due to patient confidentiality but are available from the corresponding author upon reasonable request.

## Supplementary Materials

The following supporting information can be downloaded at: https://www.mdpi.com/article/10.3390/diagnostics16020285/s1, Figure S1: Net benefit and avoided cystoscopic assessments extrapolated to 1000 urinary tests in a cohort with 260 UCBs (26.0%); Figure S2: Forest plots for sensitivity restricted to Uromonitor^®^ Version 2; Figure S3: Forest plots for specificity (a), PPV (b), NPV (c), and overall diagnostic accuracy (d) restricted to Uromonitor^®^ Version 2; Table S1: Subgroup analyses by tumor stage, tumor grade, and disease status (primary versus recurrent); Table S2: GRADE Summary of Findings; Table S3: Results of the regression-based Harbord asymmetry test assessing potential publication bias across diagnostic performance endpoints; Table S4: Reported diagnostic performance of molecular multiplex urine marker assays in non–muscle-invasive bladder cancer surveillance, based on published evidence summarized in contemporary guidelines; Table S5: Fulfilment summary for the systematic review and diagnostic accuracy meta-analysis of the Uromonitor^®^ assay and urine cytology (PRISMA 2020); Table S6: Fulfilment summary for the systematic review and diagnostic accuracy meta-analysis of the Uromonitor^®^ assay and urine cytology (PRISMA-DTA-specific items).

## References

[1] Gontero P., Birtle A., Capoun O., Compérat E., Dominguez-Escrig J.L., Liedberg F., Mariappan P., Masson-Lecomte A., Mostafid H.A., Pradere B. (2024). European Association of Urology Guidelines on Non–muscle-invasive Bladder Cancer (TaT1 and Carcinoma In Situ)—A Summary of the 2024 Guidelines Update. Eur. Urol..

[2] Holzbeierlein J.M., Bixler B.R., Buckley D.I., Chang S.S., Holmes R., James A.C., Kirkby E., McKiernan J.M., Schuckman A.K. (2024). Diagnosis and Treatment of Non-Muscle Invasive Bladder Cancer: AUA/SUO Guideline: 2024 Amendment. J. Urol..

[3] Moschini M., Gandaglia G., Dehò F., Salonia A., Briganti A., Montorsi F. (2022). Bladder cancer: ESMO Clinical Practice Guideline for diagnosis, treatment and follow-up. Ann. Oncol..

[4] Pan W., Xing J., Chen X., Xue R., Xu G., Li S., Yi H., Jin B., Wan X., Sang X. (2025). Urinary biomarkers in cancer detection: Explorations, advancements, challenges, and future directions. Int. J. Surg..

[5] Zhang D., Chen B., Ye J., Bai Y., Han P. (2025). Integrated assessment of non-invasive diagnostic tools for bladder cancer: A network meta-analysis. Front. Oncol..

[6] Wan X., Wang D., Zhang X., Xu M., Huang Y., Qin W., Chen S. (2025). Unleashing the power of urine-based biomarkers in diagnosis, prognosis and monitoring of bladder cancer (Review). Int. J. Oncol..

[7] Laukhtina E., Shim S.R., Mori K., D‘aNdrea D., Soria F., Rajwa P., Mostafaei H., Compérat E., Cimadamore A., Moschini M. (2021). Diagnostic Accuracy of Novel Urinary Biomarker Tests in Non–muscle-invasive Bladder Cancer: A Systematic Review and Network Meta-analysis. Eur. Urol. Oncol..

[8] Kravchuk A.P., Wolff I., Gilfrich C., Wirtz R.M., Soares P., Braun K.-P., Brookman-May S.D., Kollitsch L., Hauner K., Burchardt M. (2024). Urine-Based Biomarker Test Uromonitor^®^ in the Detection and Disease Monitoring of Non-Muscle-Invasive Bladder Cancer—A Systematic Review and Meta-Analysis of Diagnostic Test Performance. Cancers.

[9] Batista R., Vinagre J., Prazeres H., Sampaio C., Peralta P., Conceição P., Sismeiro A., Leão R., Gomes A., Furriel F. (2019). Validation of a Novel, Sensitive, and Specific Urine-Based Test for Recurrence Surveillance of Patients with Non-Muscle-Invasive Bladder Cancer in a Comprehensive Multicenter Study. Front. Genet..

[10] Sieverink C.A., Batista R.P.M., Prazeres H.J.M., Vinagre J., Sampaio C., Leão R.R., Máximo V., Witjes J.A., Soares P. (2020). Clinical Validation of a Urine Test (Uromonitor-V2^®^) for the Surveillance of Non-Muscle-Invasive Bladder Cancer Patients. Diagnostics.

[11] Ramos P., Brás J.P., Dias C., Bessa-Gonçalves M., Botelho F., Silva J., Silva C., Pacheco-Figueiredo L. (2025). Uromonitor: Clinical Validation and Performance Assessment of a Urinary Biomarker Within the Surveillance of Patients with Nonmuscle-Invasive Bladder Cancer. J. Urol..

[12] Azawi N., Vásquez J.L., Dreyer T., Guldhammer C.S., Al-Juboori R.M.S., Nielsen A.M., Jensen J.B. (2023). Surveillance of Low-Grade Non-Muscle Invasive Bladder Tumors Using Uromonitor: SOLUSION Trial. Cancers.

[13] Wolff I., Kravchuk A.P., Wirtz R.M., Schlomm T., Rabien A., Rong D., Hofbauer S.L., Labonté F.K., Barski D., Otto T. (2024). Real-world performance of Uromonitor^®^ in urothelial bladder cancer detection: A multicentric trial. BJU Int..

[14] Rubio-Briones J., Ramos F.G., Sánchez A.M., Abadía I.B., Rodríguez R.M., Alcaraz A., Legaz M.M., Lozano F., Martín M.A., Cardo A.L. (2025). External validation of the Uromonitor^®^-version 2 urine test as a biomarker for optimisation of non-muscle-invasive bladder cancer management. BJU Int..

[15] Ecke T.H., Meisl C.J., Schlomm T., Rabien A., Labonté F., Rong D., Hofbauer S., Friedersdorff F., Sommerfeldt L., Gagel N. (2025). Performance of Urinary Markers in Patients With Suspicious Cystoscopy During Follow-up of Recurrent Non-muscle Invasive Bladder Cancer: BTA Stat, NMP22 BladderChek, UBC Rapid Test, CancerCheck UBC Rapid VISUAL, and Uromonitor in Comparison to Cytology. Urology.

[16] Rabien A., Rong D., Rabenhorst S., Schlomm T., Labonté F., Hofbauer S., Forey N., Le Calvez-Kelm F., Ecke T.H. (2024). Diagnostic performance of Uromonitor and TERTpm ddPCR urine tests for the non-invasive detection of bladder cancer. Sci. Rep..

[17] Page M.J., McKenzie J.E., Bossuyt P.M., Boutron I., Hoffmann T., Mulrow C.D., Shamseer L., Moher D. (2020). Mapping of reporting guidance for systematic reviews and meta-analyses generated a comprehensive item bank for future reporting guidelines. J. Clin. Epidemiol..

[18] Salameh J.-P., Bossuyt P.M., A McGrath T., Thombs B.D., Hyde C.J., Macaskill P., Deeks J.J., Leeflang M., A Korevaar D., Whiting P. (2020). Preferred reporting items for systematic review and meta-analysis of diagnostic test accuracy studies (PRISMA-DTA): Explanation, elaboration, and checklist. BMJ.

[19] Tomlinson E., Cooper C., Davenport C., Rutjes A.W.S., Leeflang M., Mallett S., Whiting P. (2024). Common challenges and suggestions for risk of bias tool development: A systematic review of methodological studies. J. Clin. Epidemiol..

[20] Whiting P.F., Rutjes A.W.S., Westwood M.E., Mallett S., Deeks J.J., Reitsma J.B., Leeflang M.M.G., Sterne J.A.C., Bossuyt P.M.M., QUADAS-2 Group (2011). QUADAS-2: A revised tool for the quality assessment of diagnostic accuracy studies. Ann. Intern. Med..

[21] Schünemann H.J., Mustafa R.A., Brozek J., Santesso N., Bossuyt P.M., Steingart K.R., Leeflang M., Lange S., Trenti T., Langendam M. (2019). GRADE guidelines: 22. The GRADE approach for tests and strategies—From test accuracy to patient-important outcomes and recommendations. J. Clin. Epidemiol..

[22] Ward D.G., Gordon N.S., Boucher R.H., Pirrie S.J., Baxter L., Ott S., Silcock L., Whalley C.M., Stockton J.D., Beggs A.D. (2019). Targeted deep sequencing of urothelial bladder cancers and associated urinary DNA: A 23-gene panel with utility for non-invasive diagnosis and risk stratification. BJU Int..

[23] Eich M.-L., Pena M.D.C.R., Springer S.U., Taheri D., Tregnago A.C., Salles D.C., Bezerra S.M., Cunha I.W., Fujita K., Ertoy D. (2019). Incidence and distribution of UroSEEK gene panel in a multi-institutional cohort of bladder urothelial carcinoma. Mod. Pathol..

[24] Henning G.M., Barashi N.S., Smith Z.L. (2021). Advances in Biomarkers for Detection, Surveillance, and Prognosis of Bladder Cancer. Clin. Genitourin. Cancer.

[25] Feiertag N., Barry E., Abramson M., Park J.-Y., Kovac E., Aboumohamed A., Watts K., Sankin A. (2023). Urine Cytology Rarely Escalates Clinical Management in the Surveillance of Non–muscle-Invasive Bladder Cancer. Clin. Genitourin. Cancer.

[26] Lee J., Chen F., Lopez-Beltran A., Necchi A., Cimadamore A., Spiess P.E., Li R., Roy-Chowdhuri S., Montironi R., Golijanin D. (2025). Urinary Tumor DNA–based Liquid Biopsy in Bladder Cancer Management: A Systematic Review. Eur. Urol. Focus.

[27] Heard J.R., Mitra A.P. (2024). Noninvasive Tests for Bladder Cancer Detection and Surveillance: A Systematic Review of Commercially Available Assays. Bladder Cancer.

[28] Chang Y.-C., Peng C.-Y., Wang S.-S., Jaroenlapnopparat A., Wang J.C.H., Jou C.L., Tang P.-U., Hsia Y.P., Chiang C.-H., Chiang C.-H. (2024). Clinical performance of Bladder EpiCheck™ versus voided urine cytology for detecting recurrence of nonmuscle invasive bladder cancer: Systematic review and meta-analysis. Urol. Oncol. Semin. Orig. Investig..

[29] Sharma G., Sharma A., Krishna M., Devana S.K., Singh S.K. (2022). Xpert bladder cancer monitor in surveillance of bladder cancer: Systematic review and meta-analysis. Urol. Oncol. Semin. Orig. Investig..

[30] Bessa-Gonçalves M., Brás J.P., Jesus T.T., Prazeres H., Soares P., Vinagre J. (2024). TERTmonitor Efficacy and Performance in Detecting Mutations by Droplet Digital PCR. Genes.

