# Supplementary material for: Diagnostic Performance and Clinical Utility of the Uromonitor® Molecular Urine Assay for Urothelial Carcinoma of the Bladder: A Systematic Review and Diagnostic Accuracy Meta-Analysis
Source: Diagnostics (Basel). 2026 Jan 16;16(2):285. doi: 10.3390/diagnostics16020285 (PMC12840495; doi:10.3390/diagnostics16020285)
Supplement: Supplementary file 1 [file diagnostics-16-00285-s001.zip › diagnostics-4082381-supplementary.pdf]

Supplementary Material to the Article:

Diagnostic Performance and Clinical Utility of the Uromonitor® Molecular Urine Assay for Urothelial Carcinoma of the Bladder: A Systematic Review and Diagnostic Accuracy Meta-analysis

Julio Ruben Rodas Garzaro[1], Anton Kravchuk[1], Maximilian Burger[2], Ingmar Wolff[3], Steffen Lebentrau[4], José Rubio-Briones[5], João Paulo Brás[6-7], Christian Gilfrich[1], Stephan Siepmann[1], Sascha Pahernik[8], Axel Merseburger[9], Axel Heidenreich[10], and Matthias May[1]

1 - Department of Urology, St. Elisabeth Hospital Straubing, Brothers of Mercy Hospital, Straubing, Germany; 2 - Department of Urology, Caritas St. Josef Medical Center, University of Regensburg, Regensburg, Germany; 3 - Department of Urology, University Medicine Greifswald, Greifswald, Germany; 4 - Department of Urology, Werner Forssmann Hospital, Eberswalde, Germany; 5 - Department of Urology, Hospital VITHAS 9 de Octubre, Valencia, Spain; 6 - Instituto de Investigação e Inovação em Saúde (i3S), Universidade do Porto, Porto, Portugal; 7 - Instituto de Patologia e Imunologia Molecular (IPATIMUP), Universidade do Porto, Porto, Portugal; 8 - Department of Urology, Nuremberg General Hospital, Paracelsus Medical University, Nuremberg, Germany; 9 - Department of Urology, University Hospital Schleswig-Holstein, Campus Lübeck, Lübeck, Germany; 10 - Department of Urology, University Hospital Cologne, Cologne, Germany

Corresponding Author: Prof. Dr. Matthias May, Department of Urology, St. Elisabeth Hospital Straubing, Brothers of Mercy Hospital, Straubing, Germany
Email: matthias.may@klinikum-straubing.de

Journal: Diagnostics (MDPI)
Special Issue: Diagnostic and Prognostic Non-Invasive Markers in Bladder Cancer
Contents of this Supplementary File: Supplementary Figures 1-3 & Supplementary Tables 1-6

Supplementary Figures

Supplementary Figure 1 Net benefit and avoided cystoscopic assessments extrapolated to 1000 urinary tests in a cohort with 260 UCBs (26.0%)

Net benefit calculations and estimated reductions in cystoscopic procedures based on 1000 hypothetical tests, with 182 positive results (TP 144, FP 38) and 818 negative results (TN 702, FN 116) generated by Uromonitor®.

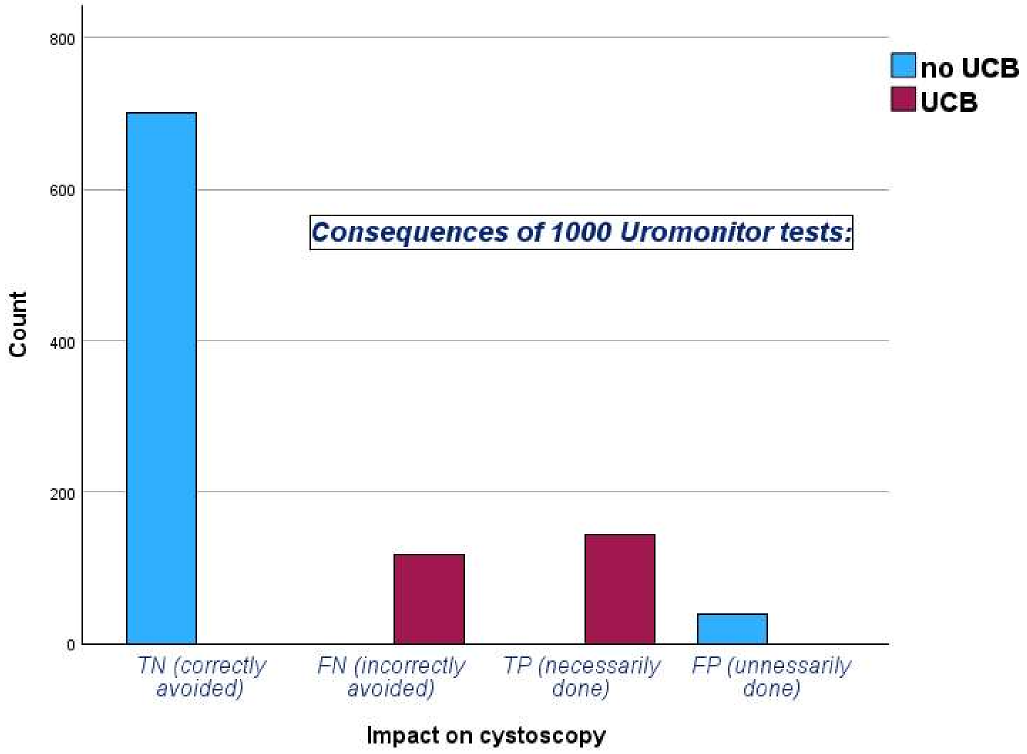

Supplementary Figure 2. Forest plots for sensitivity restricted to Uromonitor® Version 2

Forest plots showing sensitivity estimates for studies evaluating Uromonitor® Version 2 only.

Study	OR	95% CI		p	Weight	Weight(%)
Batista	50.00	5.43	460.52	0.00	0.23	11.18
Sieverink	37.80	6.49	220.22	0.00	0.26	12.54
Ecke	1.04	0.61	1.77	0.89	0.32	15.47
Rabien	0.88	0.50	1.56	0.66	0.32	15.42
Wolff	1.21	0.88	1.65	0.24	0.32	15.71
Rubio-Briones	1.30	0.74	2.29	0.36	0.32	15.42
Ramos	33.31	10.59	104.76	0.00	0.29	14.26
OR	4.26	0.75	24.29	0.09		

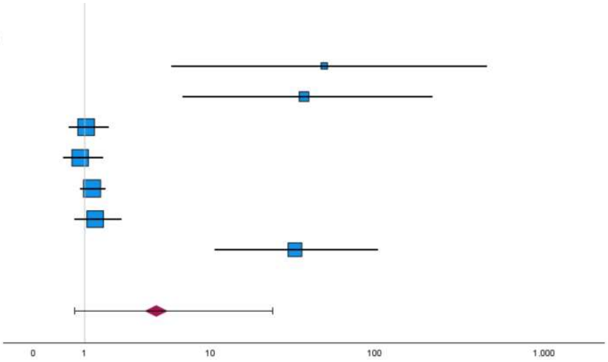

Random Effect Model
Heterogeneity: $Tau^2 = 3.07$, $H^2 = 29.89$, $I^2 = 0.97$
Homogeneity: Q = 57.46, df = 6, p-value = 0.00

Supplementary Figure 3. Forest plots for specificity (3a), PPV (3b), NPV (3c), and overall diagnostic accuracy (3d) restricted to Uromonitor® Version 2

Forest plots showing specificity, positive predictive value, negative predictive value, and overall diagnostic accuracy for studies evaluating Uromonitor® Version 2 only.

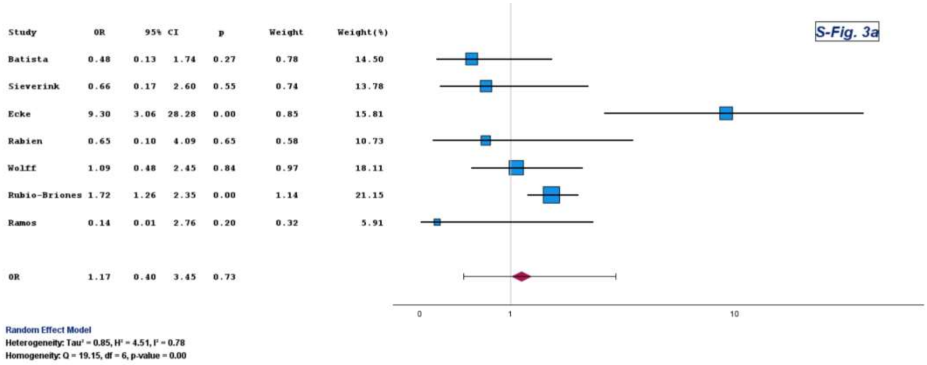

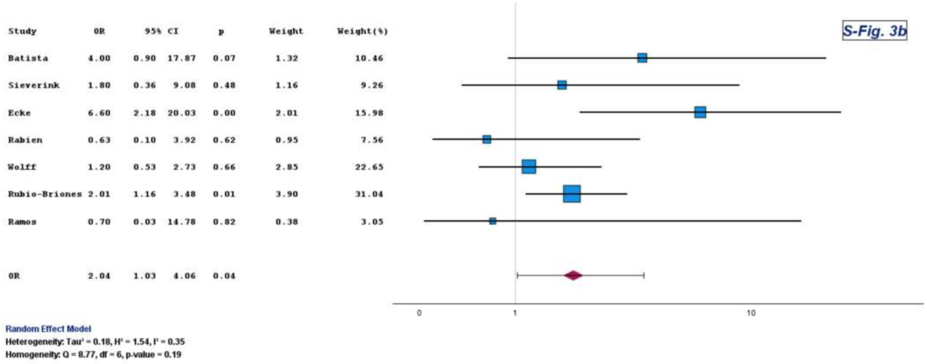

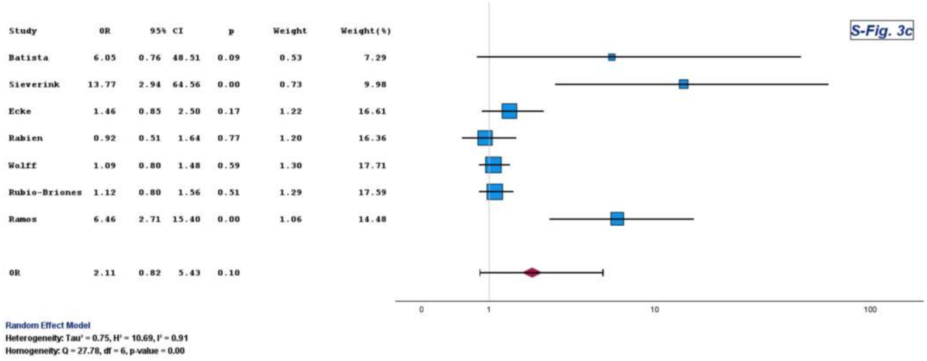

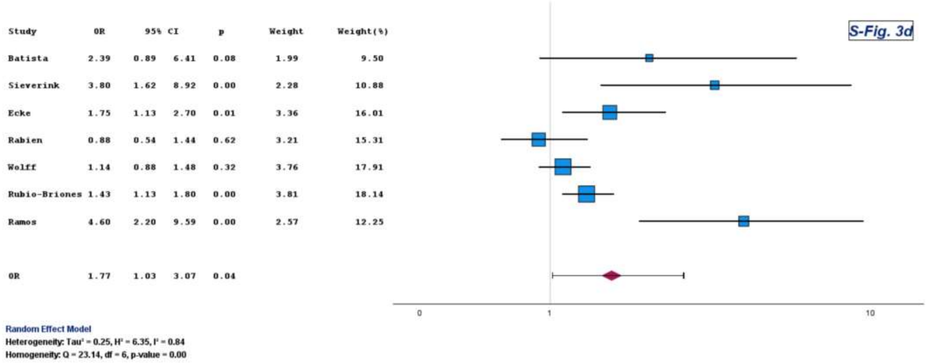

Supplementary Tables

Supplementary Table 1. Subgroup analyses by tumor stage, tumor grade, and disease status (primary versus recurrent)

Diagnostic performance of Uromonitor® and urine cytology across prespecified clinical subgroups defined by tumor stage, tumor grade, and primary versus recurrent disease.

Subgroup	Number of Studies (patients with UCB) Uromonitor® vs. Urine cytology	Pooled Sensitivity Uromonitor® vs. Urine cytology (95% CI)
**NMIBC**	7 (535) vs. 4 (415)	58.9% (54.6 – 63.1) vs. 44.1% (39.3 – 49.0)
**MIBC**	3 (85) vs. 3 (85)	56.5% (45.3 – 67.2) vs. 64.7% (53.6 – 74.8)
**LG**	6 (271) vs. 3 (220)	50.9% (44.8 – 57.0) vs. 31.8% (25.7 – 38.4)
**HG**	5 (284) vs. 3 (226)	62.7% (56.8 – 68.3) vs. 60.6% (53.9 – 67.0)
**Primary**	2 (287) vs. 2 (287)	49.5% (43.6 – 55.4) vs. 46.3% (40.5 – 52.3)
**Recurrent**	6 (306) vs. 5 (237)	64.1% (58.4 – 69.4) vs. 49.8% (43.3 – 56.3)

Legend: HG: high-grade; LG: low-grade; MIBC: muscle-invasive bladder cancer; NMIBC: non-muscle-invasive bladder cancer; UCB: urothelial carcinoma of the bladder

Supplementary Table 2. GRADE Summary of Findings

Certainty of evidence for the primary outcome (sensitivity) and the secondary outcomes (specificity, positive predictive value, negative predictive value, and accuracy) assessed using the GRADE framework for diagnostic test accuracy. Reported domains include effect sizes, precision, risk of bias, inconsistency, indirectness, publication bias, and the overall certainty ratings.

Outcome	Studies (participants Uromonitor® vs. Urine cytology)	Effect size (pooled OR with 95% CI)	Risk of bias	Inconsistency	Indirectness	Imprecision	Publication bias	Certainty of Evidence
**Sensitivity**	7 (2,816 vs. 2562)	3.15 (0.72 - 13.75) Among 1,000 patients with UCB, between 518 and 585 are correctly identified using Uromonitor® with 95% confidence, whereas urine cytology detects between 387 and 463.	Downgraded (serious) → high risk in Azawi + unclear	Serious	Not serious	Downgraded (serious) → wide CI	**Downgraded (serious)** → Harbord test p < 0.05	Given the cumulative impact of methodological limitations, substantial inconsistency driven by marked between study heterogeneity, the wide confidence interval, and the presence of publication bias, the overall certainty of evidence is rated as **very low**.
**Specificity**	7 (2,816 vs. 2562)	1.50 (0.55 - 4.04) Among 1,000 patients without UCB, between 939 and 957 are correctly classified using Uromonitor® with 95% confidence, whereas urine cytology classifies between 899 and 926.	Not serious	Not serious	Not serious	Downgraded (serious)	**Not detected**	Although the evidence is limited by imprecision, the overall certainty is rated as **moderate** given the consistency of findings and absence of serious concerns in the remaining domains.
**PPV**		2.17 (1.00 - 4.74) Among 1,000 positive test results, between 755 and 821 are accurate using Uromonitor® with 95%	Not serious	Not serious	Not serious	Downgraded (borderline)	**Not detected**	Given the borderline imprecision but absence of additional major limitations, the overall certainty of evidence is

	7 (2,816 vs. 2562)	confidence, whereas urine cytology yields between 595 and 685 correct results.						considered **moderate** based on consistency of results and adequate methodological integrity.
**NPV**	7 (2,816 vs. 2562)	1.79 (0.76 - 4.19) Among 1,000 negative test results, between 843 and 870 are accurate using Uromonitor® with 95% confidence, whereas urine cytology yields between 796 and 830 correct results.	Not serious	Not serious	Not serious	Downgraded (serious)	**Not detected**	Due to imprecision that affects confidence in the effect estimate, the overall certainty of evidence is rated as **low** despite the absence of additional serious concerns.
**Accuracy**	7 (2,816 vs. 2562)	1.72 (1.01 - 2.90) In a cohort of 1,000 patients, of whom 260 have UCB, between 833 and 856 test results are accurate using Uromonitor® with 95% confidence, whereas urine cytology yields between 766 and 798 correct results.	Not serious	Not serious	Not serious	Downgraded (borderline)	**Not detected**	Given the borderline imprecision but no additional serious limitations, the overall certainty of evidence is judged to be **moderate** based on consistent findings across studies.

Legend: CI: confidence interval; OR: odds ratio; PPV: positive predictive value; NPV: negative predictive value; UCB: urothelial carcinoma of the bladder

Supplementary Table 3. Results of the regression-based Harbord asymmetry test assessing potential publication bias across diagnostic performance endpoints

For each metric, the T value, two-sided 95% confidence interval, and *p* value are reported. Interpretation is based on the *p* value of the regression coefficient. Analyses were performed for pooled assay generations (Uromonitor® Version 1 and Version 2 combined) and for Version 2 only to ensure consistency with the current generation assay. A *p* value greater than 0.05 indicates no statistically significant small study effects and therefore no evidence of publication bias. Meta regression was conducted using a random effects model with Knapp-Hartung adjustment for standard error correction.

Diagnostic endpoint	Assay setting	T-value	95 % CI lower	95 % CI upper	*p*
**Sensitivity**	All generations	3.07	1.11	12.59	.028
**Sensitivity**	Version 2 only	3.86	2.42	12.02	.012
**Specificity**	All generations	-1.70	-5.20	1.06	.150
**Specificity**	Version 2 only	-2.10	-5.40	0.54	.089
**PPV**	All generations	-0.54	-3.52	2.31	.616
**PPV**	Version 2 only	-0.85	-3.21	1.61	.433
**NPV**	All generations	2.27	-0.59	9.58	.072
**NPV**	Version 2 only	2.42	-0.23	7.72	.060
**Accuracy**	All generations	1.95	-1.02	7.50	.108
**Accuracy**	Version 2 only	1.90	-1.02	6.76	.116

Legend: CI: confidence interval; NPV: negative predictive value; PPV: positive predictive value; V2: Version 2 of the Uromonitor® molecular assay

Supplementary Table 4. Reported diagnostic performance of molecular multiplex urine marker assays in non–muscle-invasive bladder cancer surveillance, based on published evidence summarized in contemporary guidelines

Marker / Assay	Biomarker class / Target	Reported sensitivity, % (95% CI)	Reported specificity, % (95% CI)	Key limitations	Reference - systematic review (N studies / N patients)
**Xpert BC® Monitor**	RNA-based (mRNA expression panel; 5 mRNAs)	73 (65 – 80)	77 (69 – 84)	Substantial between-study heterogeneity; variability in assay versions and diagnostic thresholds; predominantly observational study designs; absence of direct head-to-head comparisons between molecular assays.	[1] 11 studies 2,896 patients
**Bladder EpiCheck™**	DNA-based (DNA methylation panel; 15 DNA methylations)	81 (63 – 91)	87 (83 – 91)	Heterogeneous methylation cut-offs across studies; limited prospective validation in standardized surveillance settings; variability in study design and patient selection; absence of direct head-to-head comparisons between molecular assays.	[2] 6 studies, 1,588 patients
**Cxbladder Monitor®**	RNA-based (mRNA expression panel; 5 mRNAs)	91 (85 – 95)	61 (21 – 90)	Limited number of studies specifically addressing surveillance cohorts; heterogeneity across assay versions and clinical use scenarios; restricted external validation; absence of direct head-to-head comparisons between molecular assays.	[3] 2 studies, 1,112 patients
**Uromonitor®**	DNA-based (mutation panel: TERT promoter and FGFR3 in version 1; TERT promoter, FGFR3, and KRAS in version 2)	80 (n.a.)	97 (n.a.)	Marked heterogeneity across cohorts; sensitivity influenced by tumor biology and pre-analytical workflows; variability related to mutation panel composition across assay versions; absence of direct head-to-head comparisons between molecular assays.	[4] 4 studies 1,190 patients

Legend: All assays are evaluated in the context of non–muscle-invasive bladder cancer surveillance and are intended as adjuncts to cystoscopy rather than stand-alone diagnostic tests. Reported sensitivity and specificity values represent ranges derived from published systematic reviews and are based on heterogeneous study populations and differing

reference standards. Direct head-to-head comparisons between assays are lacking; therefore, reported performance metrics should not be interpreted as comparative rankings.
Abbreviations: CI, confidence interval; n.a., not available

References: [1] Sharma G, Sharma A, Krishna M, Devana SK, Singh SK. Xpert bladder cancer monitor in surveillance of bladder cancer: Systematic review and meta-analysis. Urol Oncol. 2022 Apr;40(4):163.e1-163.e9. [2] Chiang CH, Chang YC, Peng CY, Wang SS, Jaroenlapnopparat A, Wang JCH, Jou CL, Tang PU, Hsia YP, Chiang CH, Chiang CH. Clinical performance of Bladder EpiCheck™ versus voided urine cytology for detecting recurrence of nonmuscle invasive bladder cancer: Systematic review and meta-analysis. Urol Oncol. 2024 Dec;42(12):449.e21-449.e28. [3] Laukhtina E, Shim SR, Mori K, D'Andrea D, Soria F, Rajwa P, Mostafaei H, Compérat E, Cimadamore A, Moschini M, Teoh JY, Enikeev D, Xylinas E, Lotan Y, Palou J, Gontero P, Babjuk M, Witjes JA, Kamat AM, Roupret M, Shariat SF, Pradere B; European Association of Urology–Young Academic Urologists (EAU-YAU): Urothelial Carcinoma Working Group. Diagnostic Accuracy of Novel Urinary Biomarker Tests in Non-muscle-invasive Bladder Cancer: A Systematic Review and Network Meta-analysis. Eur Urol Oncol. 2021 Dec;4(6):927-942. [4] Kravchuk AP, Wolff I, Gilfrich C, Wirtz RM, Soares P, Braun KP, Brookman-May SD, Kollitsch L, Hauner K, Burchardt M, Bründl J, Burger M, May M. Urine-Based Biomarker Test Uromonitor® in the Detection and Disease Monitoring of Non-Muscle-Invasive Bladder Cancer-A Systematic Review and Meta-Analysis of Diagnostic Test Performance. Cancers (Basel). 2024 Feb 11;16(4):753.

Supplementary Table 5. Fulfilment summary for the systematic review and diagnostic accuracy meta-analysis of the Uromonitor® assay and urine cytology (PRISMA 2020)

Summary of adherence to PRISMA 2020 reporting domains for the systematic review and diagnostic accuracy meta-analysis evaluating Uromonitor® and urine cytology.

PRISMA 2020 Item	Requirement	Manuscript Location	Fulfilled
**1. Title**	Identify as a systematic review	Title page	Yes
**2. Abstract**	Structured abstract following PRISMA	Abstract	Yes
**3. Rationale**	Describe background	Introduction	Yes
**4. Objectives**	State explicit review question	Introduction	Yes
**5. Eligibility criteria**	Define inclusion and exclusion criteria	Methods: Eligibility	Yes
**6. Information sources**	All sources & dates	Methods: Search Strategy	Yes
**7. Search strategy**	Full reproducible search	Methods: Search Strategy	Yes
**8. Selection process**	Screening procedure, reviewers	Methods: Study Identification	Yes
**9. Data collection process**	Extraction process, reviewers	Methods: Data Extraction	Yes
**10a. Data items**	List outcomes extracted	Methods: Data Extraction	Yes
**10b. Other variables**	Study characteristics extracted	Methods: Data Extraction	Yes
**11. Risk of bias assessment**	QUADAS-2 procedure	Methods: Risk of Bias	Yes
**12. Effect measures**	Define diagnostic outcomes	Methods: Statistical Analysis	Yes
**13a. Synthesis methods: criteria for study eligibility for synthesis**	How studies enter meta-analysis	Methods: Statistical Analysis	Yes
**13b. Data preparation**	Handling missing/indeterminate data	Methods: Data Extraction	Yes
**13c. Risk of bias integration**	How RoB informs synthesis	Methods: Risk of Bias; GRADE	Yes
**13d. Statistical models used**	Bivariate model; rationale	Methods: Statistical Analysis	Yes
**13e. Heterogeneity exploration**	Sensitivity analyses	Methods: Sensitivity Analyses	Yes
**13f. Sensitivity analyses**	Describe planned analyses	Methods: Sensitivity Analyses	Yes
**14. Reporting bias assessment**	Methods to assess publication bias	Results: Harbord test	Yes
**15. Certainty of evidence**	GRADE for diagnostic accuracy	Methods: Risk of Bias and Applicability Assessment	Yes
**16. Study selection results**	Numbers screened/included; PRISMA flow	Results: Study Selection; Figure 1	Yes
**17. Study characteristics**	Present characteristics of included studies	Results: Study Characteristics; Table 1	Yes
**18. Risk of bias results**	QUADAS-2 outcome	Results: Risk of Bias; Figure 3	Yes
**19. Results of individual studies**	2×2 data or metrics	Supplementary Tables 2–3	Yes
**20a. Results of syntheses**	Summary accuracy estimates	Results: Diagnostic Accuracy	Yes
**20b. Heterogeneity results**	Sensitivity and heterogeneity findings	Results: Sensitivity Analyses	Yes
**20c. Reporting bias results**	Publication bias results	Results: Harbord test	Yes
**21. Overall certainty**	GRADE summary	Results: GRADE Summary; Supp. Material	Yes
**22. Discussion: Summary of evidence**	Summarise key findings	Discussion (paragraphs 1–3)	Yes
**23. Discussion: Limitations of studies**	Limitations of included evidence	Discussion (Limitations)	Yes
**24. Discussion: Limitations of review**	Limitations of methods	Discussion (Limitations)	Yes
**25. Discussion: Implications**	Clinical meaning	Discussion: Clinical Implications	Yes

**26. Funding**	Funding statement	Funding Statement	Yes
**27. Competing interests**	Conflicts of interest	Conflict of Interest Statement	Yes
**28. Data availability**	Data transparency	Data Availability Statement	Yes
**29. Registration**	Registration details	Methods: Protocol & Registration	Yes
**30. Protocol accessibility**	Whether protocol is available	Methods: Protocol	Yes
**31. Support**	Financial or non-financial support	Funding Statement	Yes
**32. Role of funder**	Influence on the review	Funding Statement	Yes
**33. Other information**	Supplements, appendices	Supplementary Materials	Yes

Supplementary Table 6. Fulfilment summary for the systematic review and diagnostic accuracy meta-analysis of the Uromonitor® assay and urine cytology (PRISMA-DTA specific items)

Summary of adherence to PRISMA-DTA specific reporting domains for the systematic review and diagnostic accuracy meta-analysis evaluating Uromonitor® and urine cytology.

PRISMA-DTA Item	Requirement	Manuscript Location	Fulfilled
**Title**	Identify as diagnostic test accuracy systematic review	Title page	Yes
**Abstract**	Structured abstract including index test, target condition, methods, results	Abstract	Yes
**Rationale**	Describe clinical and methodological rationale	Introduction (paragraphs 1–3)	Yes
**Objectives**	State explicit review objectives	Introduction (final paragraph)	Yes
**Protocol & Registration**	Provide protocol details and registration number	Methods: Protocol and registration	Yes
**Eligibility Criteria**	Define population, index test, comparator, target condition, study designs	Methods: Eligibility criteria	Yes
**Information Sources**	List all databases and search dates	Methods: Search strategy	Yes
**Search Strategy**	Present reproducible search string	Methods: Search Strategy	Yes
**Study Selection**	Describe screening process and flow	Methods: Study identification; Figure 1	Yes
**Data Collection Process**	Describe extraction procedure and reviewers	Methods: Data extraction	Yes
**Data Items**	List variables extracted (thresholds, setting, reference standard)	Methods: Data extraction	Yes
**Risk of Bias Assessment**	QUADAS-2 domains and procedure	Methods: Risk of bias and applicability assessment	Yes
**Applicability Concerns**	QUADAS-2 applicability assessment	Methods: same section	Yes
**Diagnostic Thresholds**	Define thresholds; harmonisation of cytology (Paris binary); positivity rule for Uromonitor®	Methods: Index Test Definition; Comparator Definition	Yes
**Rationale for Meta-analysis Methods**	Justification for bivariate model; HSROC rationale	Methods: Statistical analysis	Yes

**Investigations of Heterogeneity**	Prespecified factors; sensitivity analyses	Methods: Sensitivity analyses	Yes
**Handling of Indeterminate Results**	Describe handling of equivocal or uninterpretable tests	Methods: Data extraction	Yes
**Missing Test Data**	Describe handling of missing cytology or molecular data	Methods: Data extraction	Yes
**Zero-Cell Handling**	Specify whether zero cells occurred and how handled	No zero-cell tables; no correction needed	Yes
**Statistical Synthesis**	Describe models, parameters, CIs, software	Methods: Statistical analysis	Yes
**Additional Analyses**	Sensitivity analyses, version comparisons	Methods & Results: Sensitivity analyses	Yes
**Study Selection Results**	Report numbers screened and included	Results: Study selection; Figure 1	Yes
**Study Characteristics**	Describe patient cohorts, settings, Uromonitor® versions	Results: Study characteristics; Table 1	Yes
**Risk of Bias Results**	Present QUADAS-2 outcomes	Results: Risk of bias; Figure 3	Yes
**Individual Study Results**	Provide 2×2 tables or test metrics	Table 1, Supplementary table 1	Yes
**Synthesis of Results**	Provide pooled sensitivity, specificity, PPV, NPV, accuracy	Results: Diagnostic accuracy	Yes
**Additional Analyses Results**	Present findings from sensitivity and heterogeneity analyses	Results: Sensitivity analyses; Supplementary table 1	Yes
**Summary of Evidence**	Summarise principal findings	Discussion (paragraphs 1–3)	Yes
**Limitations**	Limitations of evidence and review process	Discussion (limitations paragraph)	Yes
**Conclusions**	Clinical implications and integration into pathways	Discussion: Clinical implications	Yes
**Funding**	Report funding and role of funder	Funding statement	Yes
